# A Review on Protocatechuic Acid and Its Pharmacological Potential

**DOI:** 10.1155/2014/952943

**Published:** 2014-03-26

**Authors:** Sahil Kakkar, Souravh Bais

**Affiliations:** Department of Pharmacology, Rayat Institute of Pharmacy, Railmajra, District S.B.S. Nagar, Punjab 144533, India

## Abstract

Flavonoids and polyphenols are heterocyclic molecules that have been associated with beneficial effects on human health, such as reducing the risk of various diseases like cancer, diabetes, and cardiovascular and brain diseases. Protocatechuic acid (PCA) is a type of widely distributed naturally occurring phenolic acid. PCA has structural similarity with gallic acid, caffeic acid, vanillic acid, and syringic acid which are well-known antioxidant compounds. More than 500 plants contain PCA as active constituents imparting various pharmacological activity and these effects are due to their antioxidant activities, along with other possible mechanisms, such as anti-inflammatory properties and interaction with several enzymes. Over the past two decades, there have been an increasing number of publications on polyphenols and flavonoids, which demonstrate the importance of understanding the chemistry behind the antioxidant activities of both natural and synthesized compounds, considering the benefits from their dietary ingestion as well as pharmacological use. This work aims to review the pharmacological effects of PCA molecules in humans and the structural aspects that contribute to these effects.

## 1. Introduction

**Figure 1:**
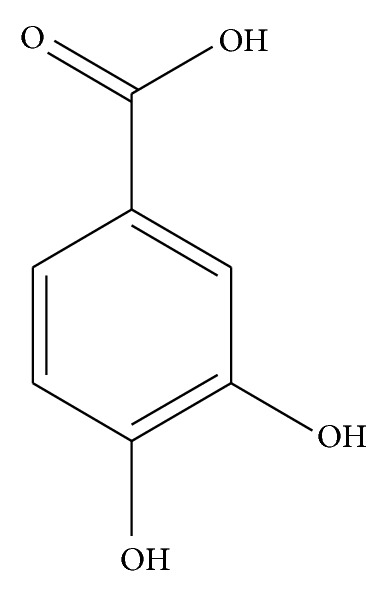
Chemical Structure of protocatechuic acid.

Polyphenols are the most voluminous antioxidants in human diets. These polyphenols are to be categorized in different classes as phenolic acids, flavonoids, lignans, and stilbenes. Phenolic acids are naturally occurring compounds found in plant kingdom with unique structural similarities, presence of carboxylic group as in caffeic acid, gallic acid, p-coumaric acid, vanillic acid, ferulic acid, and protocatechuic acid (PCA) [1] (Figure 1). Protocatechuic acid (PCA) is widely distributed and present in most edible plants used in folk medicine [2]. It is also a very common compound present in human diet, present in bran and grain brown rice (Oryza sativa L.) [3] and onion (*Allium cepa* L.) [4], especially in the scales. Protocatechuic acid is detected in many fruits, such as plums (*Prunus domestica* L.) [5]; gooseberries (*Ribes uva-crispa* L.) [6]; grapes (*Vitis vinifera*) [6]; and nuts, such as almonds ordinary (*Prunus amygdalus*) [7]. It is present in products of plant origin, such as olive oil or white wine [8, 9]. Protocatechuic acid is also found in many plants and spices, such as star anise (*Illicium verum*), melissa (*Melissa officinalis* L.), a medical rosemary (*Rosmarinus officinalis* L.), and cynamonowa (*Cinnamomum aromaticum*) [4]. This compound is one of the biologically active components of some medicinal plants, including those used in natural medicine, such as sudan Mallow (*Hibiscus sabdariffa* L.) [10, 11], Japanese ginkgo (*Ginkgo biloba* L.) [12], and St. John's wort (*Hypericum perforatum* L.) [13]. PCA has been reported for its potential action such as antioxidant activity, antibacterial activity, anticancer activity, antiulcer activity, antidiabetic activity, antiageing activity, antifibrotic activity, antiviral activity, anti-inflammatory activity, analgesic activity, antiatherosclerotic activity, cardiac activity, hepatoprotective activity, neurological and nephro protective activity. Complete information about the PCA has been collected from various books, journals, and so forth. Journals of the last 20 years were searched. Particulars of pharmacological activities, phytochemical isolation, toxicity studies, and so forth. were extracted from the published reports focusing on the safety profile of the PCA. Safety of the whole plant was concluded in the review.

## 2. Protocatechuic Acid

Protocatechuic acid (PCA) is a type of widely distributed naturally occurring phenolic acid. PCA has structural similarity with gallic acid, caffeic acid, vanillic acid, and syringic acid which are well-known antioxidant compounds.

## 3. Distribution and Occurrence

Protocatechuic acid occurs in pigmented onion scales* Allium cepa* [14] which enables them resist onion smudge, a fungal disease due to* Colletotrichum circinans*. Hibiscus protocatechuic acid (PCA) is a simple phenolic compound isolated from the dried flowers of* Hibiscus sabdariffa* L. [15], a Chinese herbal medicine, which is reported to be antiseptic [16], aphrodisiac, astringent, cholagogue, demulcent, digestive, diuretic, emollient, purgative [17], refrigerant, resolvent, sedative, stomachic, and tonic. Also this is a folk remedy for abscesses, bilious conditions, cancer, cough, debility, dyspepsia, dysuria, fever, hangover, heart ailments, hypertension [18], neurosis, scurvy, and strangury. Protocatechuic acid is also found in Carrot (*Daucus carota*) and in mushrooms such as* Agaricus bisporus* (White Button Mushroom) or* Phellinus linteus* and has shown good chemopreventive properties.

Protocatechuic acid is considered as an active component of some traditional Chinese herbal medicines such as* Cibotium barometz* (L.) [19]* J.S, Stenoloma chusanum* (L.)* Ching, Ilex chinensis Sims*. Fruits of Ficus species are rich source of polyphenolic compounds and flavanoids which are responsible for strong antioxidant properties that help in prevention and therapy of various oxidative stress related diseases such as neurodegenerative and hepatic diseases. Acai oil, obtained from the fruit of the Acaí palm (*Euterpe oleracea*) [20], is rich in protocatechuic acid (630 ± 36 mg/kg). Acai oil has a relatively high content of polyphenols, which in turn has been linked to a range of reported (mostly* in vitro*) antioxidant, anti-inflammatory, antiproliferative, and cardioprotective properties. PCA also occurs in rich quantity in various multiple fruits such as berries (raspberry, blueberry, mulberry, strawberry, cranberry, and gooseberry). It is also known to occur in Loquat fruit, wine, honey, and soybean.

## 4. Chemical Properties

PCA is chemically known as 3,4-dihydroxybenzoic acid. It is a phenolic compound naturally occurring in various plant species. Phenolic compounds are considered secondary metabolites and are derived from phenylalanine via the shikimic acid pathway. Phenolics possess an aromatic ring and have one or more hydroxyl groups. Plants contain a large variety of phenolic derivatives, including benzoic acids, cinnamic acid derivatives, flavonoids, isoflavonoids, lignans, and tannins. In plants the main phenolic classes are hydroxyl benzoic acids, hydroxycinnamic acids, flavonols, anthocyanins, flavan-3-ols and proanthocyanidins, and ellagitannins.

## 5. Physical Properties

PCA is a gray to tan solid crystalline powder, with a 221°C melting point and 410°C boiling point at 760 mm Hg. It has a mild phenolic odour. It is sparingly soluble in water (1 : 50), soluble in alcohol, ether and discolors in air. PCA is generally stable but incompatible with strong oxidizing agents and strong bases. It irritates lungs, eyes, and skin.

## 6. Pharmacological Properties

A variety of research work has been carried out on protocatechuic acid, its derivatives, and coforms (esters, aldehydes, etc.). It has been found useful for treatment and/or prophylaxis for a large number of various ailments associated with oxidative stress damage in multiple body systems* in vitro* and* in vivo*.

### 6.1. Antibacterial Activity [15]

Roselle calyx (*Hibiscus sabdariffa *L.) [15] extract and protocatechuic acid were both found to decrease lipid oxidation levels in ground beef tissue. Protocatechuic acid also exhibited dose-dependent effect. The addition of roselle calyx extract or protocatechuic acid did not affect cooking loss, pH value, sensory attributes and content of fat, protein, and moisture of beef samples during storage at 4°C for 15 days. Roselle calyx also shows the presence of protocatechuic acid. These data support that roselle calyx extract and protocatechuic acid may be used for muscle foods to prevent contamination from campylobacter and aerobes and delay lipid oxidation and also serve as a good food preservative.

### 6.2. Antioxidant Activity [19]

Protocatechuic acid and other structurally similar dihydroxy and trihydroxyphenolic acids, 3,4-dihydroxyphenylacetic acid, hydrocaffeic acid, caffeic acid, gallic acid, 3,4,5-trihydroxyphenylacetic acid, 3-(3,4,5-trihydroxyphenyl) propanoic acid, and 3-(3,4,5-trihydroxy-phenyl) propanoic acid were examined for their total antioxidant capacity. 3, 4, 5-trihydroxy-phenylacetic acid showed to be most potent radical scavenger generated by AAPH in liposomes. In the lipid peroxidation assay 3, 4-dihydroxyphenylacetic acid was observed to be the most effective compound.

**Figure 2:**
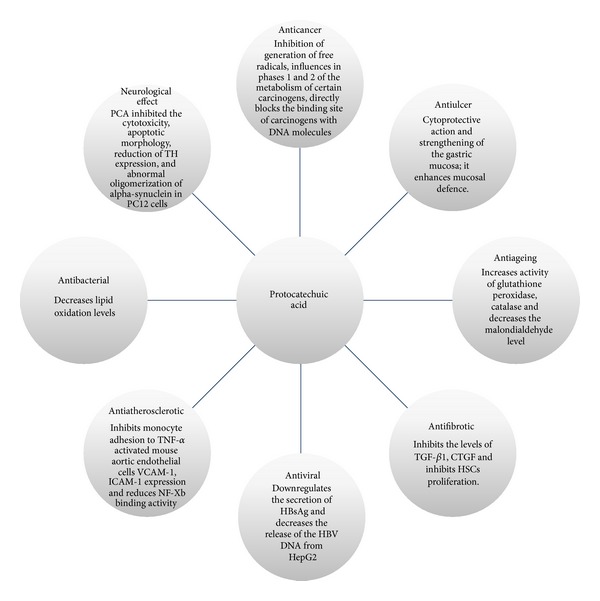
Pharmacological activity of protocatechuic acid.

The antioxidant activities of protocatechuic acid [19] were measured* in vitro *using various antioxidant assays including 1,1-diphenyl-2-picryl-hydrazyl (DPPH^•^), 2,2′-azino-bis(3-ethylbenzthiazoline-6-sulfonic acid) (ABTS^+•^), superoxide anion radicals (O_2_^−•^) and hydroxyl radical (^•^OH) scavenging activity, ferric ions (Fe^3+^) and cupric ions (Cu^2+^) reducing power, ferrous ions (Fe^2+^), and cupric ions (Cu^2+^) chelating activity, compared with the positive controls Trolox or BHT (Figure 2). PCA along with positive controls exhibited dose-dependently antioxidant ability. Comparing to a standard antioxidant Trolox, the relative antioxidant activity of PCA (i.e., the ratio of IC50 (Trolox)/IC50 (PCA)) was calculated as 2.8, 2.3, 3.7, 6.1, 4.2, 1.0, 2.7, and 1.5, respectively, for DPPH, ABTS, reducing power (Fe^3+^), reducing power (Cu^2+^), superoxide anion radical-scavenging, hydroxyl radical-scavenging, chelating ability (Fe^2+^), and chelating ability (Cu^2+^). Comparing to Trolox, PCA shows much more effective antioxidant activity* in vitro *in both lipid and aqueous media. Hence, it could therefore be used in pharmacological or food industry as a natural antioxidant. It may exhibit antioxidant activity by both chelating metal transition ions as well as by scavenging free radicals via donating hydrogen atom (H^•^) or electron (e).

Protocatechuic acid PCA is considered as an active component of some traditional Chinese herbal medicines such as* Cibotium barometz* (L.) [19] J.Sm,* Stenoloma chusanum* (L.)* Ching*,* Ilex chinensis *Sims (Table 1). PCA was reported to possess various pharmacological effects which may be closely correlated with its antioxidant activities. Hibiscus PCA supplementation was found to be beneficial in enhancing antioxidant status and inhibiting oxidative stress induced by exhaustive exercise in skeletal muscles.

### 6.3. Antidiabetic Activity [29]

Protocatechuic acid at 1% and 2% when given to d-galactose treated mice for 8 weeks decreased reactive oxygen species levels, protein carbonyl, carboxymethyllysine, pentosidine, sorbitol, fructose, and methylglyoxal. PCA also shows anti-inflammatory properties in this regard by decreased release of interleukin (IL)-1 beta, tumor necrosis factor-alpha, and prostaglandin E2 in brain. Protocatechuic acid might be helpful for the prevention or alleviation of ageing due to prevention of brain inflammatory and glycative injury. PCA at 2% or 4% when supplied to diabetic mice for 12 weeks was useful in preventing glycation-associated diabetic complications.

In other study cyanidin-3-O-*β*-glucoside [29] and PCA have been proposed to exert insulin-like activities by PPAP*γ* activation, evidencing a causal relationship between this transcription factor and adiponectin and GLUT4 upregulation. Thus PCA may be a promising antidiabetic agent for the future.

### 6.4. Anticancer Activity [30]

PCA seems to have chemopreventive potential because it inhibits the* in vitro* chemical carcinogenesis and exerts proapoptotic and antiproliferative effects in different tissues (Table 2). The mechanism of the chemopreventive action of protocatechuic acid is mostly associated with antioxidant activity, including inhibition of generation as well as scavenging of free radicals and upregulating antioxidant enzymes. It influences phases 1 and 2 of the metabolism of certain carcinogens and, perhaps, directly blocks specific binding sites of ultimate carcinogens with DNA molecule, thus preventing adduct formation that may result in mutations and neoplastic transformation. Other biological aspects seem to have influence on the activity of inducible isoenzyme of cyclooxygenase and nitric oxide synthase, cell cycle regulating proteins, or inflammatory cytokines, which are involved in oncogenesis. Thus PCA seems to have potential cancer chemopreventive properties.

### 6.5. Antiulcer Activity [31]

Protocatechuic acid ethyl ester was studied in rats in which gastric ulcers were induced by oral administration of ethanol or aspirin or by pyloric ligation. PCA ethyl ester administered at the dose of (30 mg/kg and 60 mg/kg i.p.) 30 min prior to ulcer induction was found to possess significant antiulcer property and the ulcer index was significantly less in comparison to vehicle control animals. The mechanism of action of PCA ethyl ester may be due to either cytoprotective action of the drug or by strengthening the gastric mucosa thereby enhancing mucosal defense. Similarly protocatechuic acid may also possess a certain level of antiulcer properties.

### 6.6. Antiageing Activity [32]

Protocatechuic acid derived from the dried fruits of* Alpinia oxyphylla* has proved to be a potential antiageing compound on spleen and liver antioxidant system in aged rats. Young and old rats were treated with single doses of Alpinia PCA (5 mg/kg (low dose) or 10 mg/kg (high dose) i.p. for 7 days). The results proved that Alpinia PCA significantly elevated the splenic weights, increased the activities of glutathione peroxidase and catalase, and decreased the malondialdehyde level of aged rats. Thus PCA may be therapeutically utilized to minimize age-associated disorders where oxidative damage is the major cause.

### 6.7. Antifibrotic Activity [33]

Studies have shown protocatechuic aldehyde to possess beneficial antifibrogenic effects. Transforming growth factor-*β*1 (TGF-*β*1) and connective transforming growth factor (CTGF) are associated with the pathophysiology of liver fibrosis. In carbon tetrachloride (CCL_4_) induced rat liver fibrosis model, liver fibrosis grade, and histopathological changes were evaluated, and biochemical indicators were determined. Protocatechuic aldehyde was seen to inhibit the levels of TGF-*β*1, CTGF inhibit HSCs proliferation, type I collagen, and type III collagen in TNF-*α* stimulated HSCs. Also it causes significant reduction in fibrosis grade, ameliorates biochemical indicators, and histopathological morphology and reduces liver TGF-*β*1 and CTGF expression.

### 6.8. Antiviral Activity [34]

Protocatechuic aldehyde derived from the Chinese herb,* Salvia miltiorrhiza,* has been reported to inhibit hepatitis B virus (HBV) replication in HepG2 2.2.15 cell line* in vitro* and duck hepatitis B virus (DHBV) replication in duckling's* in vivo*. Protocatechuic aldehyde's mechanism seemed to downregulate the secretion of HBsAg and HBeAg and decrease the release of HBV DNA from HepG2 2.2.15 in a dose- and time-dependent manner occurring at concentrations between 24 and 48 g/mL. Also protocatechuic aldehyde when given (25, 50, or 100 mg/kg, i.p. twice daily) also reduced viremia in DHBV-infected ducks. This activity tells us that structurally similar compound protocatechuic acid may also possess certain levels of antiviral activity and can be an effective antiviral agent.

### 6.9. Anti-Inflammatory, Analgesic [35], and Antiseptic Properties

Protocatechuic acid has shown promising anti-inflammatory and analgesic activity in different rat models (carrageenan-induced paw oedema, cotton pellet-induced granuloma, and Freund's adjuvant arthritis) [35] of inflammation and chemical and heat induced mouse models of pain. Treatment with PCA inhibits significantly different biological parameters like hind paw oedema, granuloma exudates formation, and arthritis index in carrageenan oedema, cotton pellet granuloma, and Freund's adjuvant arthritis, respectively. The biochemical parameters like glutathione, superoxide dismutase, catalase, lipid peroxidation and NO in oedematous or in liver tissues and serum alanine aminotransferase, and lactic dehydrogenase occurring during different types of inflammation were either significantly restored or inhibited with PCA pretreatment.

Reference [2] in other study anthocyanins and their breakdown metabolites, protocatechuic, syringic, gallic, and vanillic acids were evaluated on different parameters involved in atherosclerosis, including inflammation, cell adhesion, chemotaxis, endothelial function, estrogenic/antiestrogenic activity and angiotensin-converting enzyme (ACE) inhibitor activity. Protocatechuic acid was found to exhibit a slight inhibitory effect on NO production and TNF-a secretion in LPS-INF-c-induced macrophages. All anthocyanins showed an ACE-inhibitory activity.

PCA has also displayed its anticoagulant, anti-inflammatory, and antioxidative effects in diabetic mice. PCA at 1%, 2%, and 4% was given to diabetic mice for 8 weeks which significantly lowered plasma glucose and increased insulin levels. Also PCA treatments at 2% and 4% significantly lowered plasminogen activator inhibitor-1 activity and fibrinogen level; increased plasma activity of antithrombin-III and protein C; decreased triglyceride content in plasma, heart, and liver; elevated glutathione level and the retention of glutathione peroxidase and catalase activities in heart and kidney. PCA treatments also reduced the levels of interleukin-6 and tumor necrosis factor-*α* in heart and kidney. Thus PCA could be highly useful in diabetic complications via its triglyceride-lowering, anticoagulatory, antioxidative, and anti-inflammatory effects.

### 6.10. Antiatherosclerotic and Hyperlipidemic Activities [36]

Protocatechuic acid (PCA) has been found to possess the antiatherosclerotic effect. PCA inhibits monocyte adhesion to tumor necrosis factor-*α* (TNF-*α*)-activated mouse aortic endothelial cells, which is associated with the inhibition of vascular cell adhesion molecule 1 (VCAM-1) and intercellular adhesion molecule 1 (ICAM-1) expression and reduces NF-*κ*B binding activity. PCA possesses the antiatherogenic effect by virtue of its anti-inflammatory activity.

Protocatechuic aldehyde (PA), isolated from the aqueous extract of the root of* Salvia miltiorrhiza*, an herb used in traditional Chinese medicine which has been used to treat a variety of vascular diseases, was tested on the migration and proliferation of VSMCs and platelets due to platelet-derived growth factor (PDGF). DNA 5-bromo-2′-deoxy-uridine (BrdU) incorporation and wound-healing assays indicated that PA significantly attenuated PDGF-induced proliferation and migration of VSMCs at a pharmacologically relevant concentration (100 *μ*M). On a molecular level, it was observed downregulation of the phosphatidylinositol 3-kinase (PI3K)/Akt and the mitogen-activated protein kinase (MAPK) pathways, both of which regulate key enzymes associated with migration and proliferation.

The ethanolic polyphenolic extracts of* H. sabdariffa* possess significant antioxidant and hyperlipidemic activities. They have shown promising effects on decrease of serum total cholesterol, VLDL-C, LDL-C, LDL-C:HDL-C risk ratio, and atherogenic index in rats. Thus PCA which is a rich polyphenolic constituent of* H. sabdariffa* may serve as a good hyperlipidemic agent.

### 6.11. Cardiac Activity

Protocatechuic acid present in the aqueous extract of petals of* Hibiscus sabdariffa* exhibited antihypertensive and cardioprotective effects in established stages of 2-Kidney, 1-Clip renovascular hypertension model in rats. This study supports the traditional use of* Hibiscus sabdariffa* exhibiting antihypertensive and cardioprotective effects and may be a useful antihypertensive agent.

*Salvia miltiorrhiza* [25] has long been used in the traditional Chinese formulations for the treatment of heart ischemic diseases. Protocatechuic acid is its major chemical constituent. Isoproterenol induced acute myocardial infarction in rats showed positive treatment effects with the extracts of* Salvia miltiorrhiza* [26] (29.76 or 59.52 mg/kg). Isoproterenol-treated rats showed reductions in left ventricular systolic pressure as well as in maximum and minimum rate of developed left ventricular pressure, together with an increase in left ventricular end-diastolic pressure. Also an increase in serum levels of lactate dehydrogenase, glutamic oxaloacetic transaminase, creatine kinase, and malondialdehyde was seen and decrease in serum activities of glutathione peroxidase and superoxide dismutase was observed.

2,3,7,8-Tetrachlorodibenzo-*p*-dioxin (TCDD) [37] cardiotoxicity in 3-4 months old rats was studied and protocatechuic acid treatment at the dose of 100 mg/kg for 45 days was found to decrease the levels of TBARS, while increasing those of glutathione, catalase, glutathione peroxidase, and superoxide dismutase. Also PCA prevented histopathological alterations such as necrosis and hemorrhage in heart tissue induced by TCDD. PCA has also shown beneficial effects in acute myocardial infarction with propranolol in dogs.

### 6.12. Hepatoprotective Activity [23]

Hibiscus protocatechuic acid (PCA), a simple isolated from* Hibiscus sabdariffa* L. was found to be protective against oxidative damage induced by tert-butylhydroperoxide (t-BHP) in a primary culture of rat hepatocytes due to its antioxidant mechanism of action (Tseng et al., 1996).* Hibiscus sabdariffa* L. PCA possesses free radical-scavenging capacity and protects against oxidative damage induced by tert-butylhydroperoxide (t-BHP) in rat primary hepatocytes. PCA (50–100 mg/kg) by gavage for 5 days inhibited t-BHP-induced tyrosine phosphorylation in the liver and was found to be effective against t-BHP-induced hepatotoxicity by means of its antioxidant and anti-inflammatory characteristics accompanied by blocking of stress signal transduction.

Alpinia PCA isolated from the dried fruits of* Alpinia oxyphylla* Miq. at the doses of 5–10 mg/kg (i.p.) for 7 days in young and old rats was found to possess antiageing effects. It significantly elevated the splenic weights, increased the activities of GSH-PX and CAT, and decreased the MDA level of aged rats. Thus PCA was thought to be therapeutically useful to minimize age-associated disorders where oxidative damage is the major cause.

### 6.13. Nephroprotective Activity [38]

The aqueous extracts from* Hibiscus sabdariffa* richly comprising protocatechuic acid possess a potent protective effect against the oxidative stress induced by sublethal dose of Malathion (an organophosphorus pesticide on the adult male rat kidney). Aqueous extract in a daily dose of 500 mg/kg b.wt./day decreased the oxidative stress levels, prevented cellular degeneration and necrosis of the renal tissues. Also serum urea and creatinine were decreased and GSH and SOD levels were also increased significantly. This study proves the utility of PCA in preventing damage by agents causing oxidative stress mediated nephrotoxicity.

*Rhus verniciflua* Stokes (RVS) [38] containing flavonoids have antioxidant effects. Protocatechuic acid is also a major phenolic acid present in* Rhus verniciflua* Stokes. The cytotoxic and nephroprotective effects of RVS were evaluated* in vitro* in cisplatin treated Madin—Darby Canine kidney (MDCK)-I renal cells. Also its* in vivo* effects were studied in BALB/c mice inoculated with CT-26 colon adenocarcinoma cells and treated with cisplatin. RVS prevented cisplatin-induced cytotoxicity and ROS release against MDCK-I cells. RVS also exerted significant antitumor activity against CT-26 cells. The serum and kidney parameters were improved, which suggests that protocatechuic acid present in RVS can be isolated and usefully applied to the neoplastic patients as a combined chemopreventive agent with cisplatin.

PCA is structurally 3,4-dihydroxy benzoic acid and its structurally similar analogue and antioxidant 2,3-dihydroxybenzoic acid (DHB) reverses the vancomycin-induced nephrotoxicity in rats. Vancomycin-induced nephrotoxicity involves oxidative injury due to free radical formation. It can be suggested that PCA could also show the same efficacy in prevention of nephrotoxicity similarly.

### 6.14. Neurological Effects [28]

Protocatechuic acid (PCA) isolated from the kernels of* Alpinia oxyphylla* protected from oxidative stress induced neurotoxicity due to hydrogen peroxide apoptosis in cultured PC12 cells. It was also found to play crucial role in the proliferation and neuroprotection of cultured neural stem and progenitor cells. PCA induced neuronal maturation and efficiently promoted neurite outgrowth.

Protocatechuic acid also showed positive effects on PC12 cells treated with MPP(+) by inhibition of the oligomerization of alpha-synuclein which affects neuronal viability. PCA inhibited the cytotoxicity, apoptotic morphology, reduction of TH expression, and abnormal oligomerization of alpha-synuclein in PC12 cells. MPP+ (1-methyl-4-phenylpyridinium ion) has also been found to cause apoptosis in dopaminergic PC12 cells. Protocatechuic acid present in ethyl acetate extract of* Alpinia oxyphylla* was found to possess neuroprotective activity against 1 methyl-4-phenylpyridinium ion (MPP+) induced apoptosis and oxidative stress in cultured PC12 cells in a dose-dependent manner. Thus PCA may find a valuable use in management of Parkinson's disease.

The neurotrophic effects of protocatechuic acid on neurite outgrowth and survival in cultured newborn rat cerebral cortical neurons was determined and it was found out that PCA increased the number of survival neurons with neurites and the average length of neuritis.

Protocatechuic acid methyl ester isolated from fraction of the bark of* Machilus thunbergii* Sieb. et Zucc. (Lauraceae) was found to possess significant neuroprotective activities against glutamate-induced neurotoxicity in primary cultures of rat cortical cells at concentrations ranging from 0.1 microM to 10.0 microM and were comparable to MK-801 which is a well-known inhibitor of glutamate receptor.

### 6.15. Effects on Reproductive System [39]

Protocatechuic acid (1 mg/kg, p.o. for 45 days) administration in rats was found to be highly beneficial in protecting against reproductive toxicity caused by 2,3,7,8-tetrachlorodibenzo-p-dioxin (TCDD), an environmental contaminant. TCDD (2 ug/kg per week) caused oxidative stress damage via an increase in the levels of TBARS and decrease in the levels of glutathione, catalase, and SOD. It also caused testicular damage, decreased the serum testosterone levels, and reduced the sperm motility and sperm count. Treatment with PCA caused a significant reversal of the oxidative, hormonal, histopathological, and spermatological parameters.

## 7. Metal Chelating Properties of PCA

The chelating abilities of protocatechuic acid on Fe^2+^ and Cu^2+^ were evaluated and compared with the positive controls, Trolox, and Butylated Hydroxy Toluene (BHT). The metal chelating activity of protocatechuic acid solution was found to be concentration-dependent and showed distinctly higher chelating activity.

In another study PCA was studied along with five phenolic acids (cynarin, caffeic, chlorogenic, ferulic, and rosmarinic acid) and was proved to possess Fe^2+^ and Cu^2+^ chelating and DPPH radical-scavenging abilities. All phenolics including PCA were found to suppress cell membrane damage induced by transition metals or tBH. The protectivity correlated with their capacity to bind transition metals and DPPH radical-scavenging ability.

## 8. Toxicity Profile of PCA

The LD_50_ of PCA was found to be 800 mg/kg by i.p. route and 3.5 g/kg by i.v. route in mice. The LD_50_ of protocatechuic aldehyde in mice by oral route was reported to be 1.7 g/kg. Toxic oral dose of 500 mg/kg in mice is known to cause depletion of GSH in liver and kidney but no mortality. Due to low absorption by oral route, PCA is a nontoxic and a relatively safe compound for oral administration.

## 9. Conclusion

The above paper reveals that the PCA is safer at its therapeutic dose of 100 mg/kg. The compound was found to be potent antioxidant, antibacterial, anticancer, antihyperlipidemic, antidiabetic, and anti-inflammatory. However, further series of studies are required to prove its clinical reliability, safety, and efficacy.

## Figures and Tables

**Table 1:** Different biological sources and uses of protocatechuic acid.

Sr. number	Biological source	Uses	Reference
1	*Oryza sativa* (brown rice)	Cancer chemopreventive	[3]
2	*Allium cepa *(Onion)	Antifungal	[4, 21]
3	*Cinnamomum aromaticum *	Antioxidant	[4]
4	*Prunus domestica *L. (plums)	Antioxidant	[5]
5	*Ribes uva-crispa *L. (*gooseberries*)	Antioxidant	[6]
6	*Vitis vinifera * (grapes)	Antioxidant	[6]
7	*Prunus amygdalus *(almond)	Antioxidant	[7]
8	*Hibiscus sabdariffa *(Roselle)	Antibacterial, Nephroprotective activity	[10, 11, 15]
9	*Ginkgo biloba *L. (ginkgo)	Antioxidant	[12]
10	*Hypericum perforatum *L*. *	Antioxidant	[13]
11	Human metabolite of* Cyanidin Glycosides *	Antioxidant	[14]
12	*Boswellia dalzielii *	Antispasmodic	[22]
13	*Cibotiumbarometz *	Antioxidant	[19]
14	*Euterpe oleracea* (Acai)	Antioxidant, anti-inflammatory, antiproliferative, and cardioprotective	[20]
15	*Hibiscus sabdariffa *	Antihypertensive, hepatoprotective, and anti-inflammatory	[23]
16	*Agaricusbisporus *or* Phellinuslinteus* (Mushrooms)	Chemopreventive properties	[24]
17	*Hedera helix * (common ivy)	Bronchodilatatory, antispasmodic activity	[24]
18	*Salvia miltiorrhiza *	Antiviral, antiatherosclerotic, hyperlipidemic, and ischemic heart disease protective	[25] [26]
19	Fruit of* Phyllanthus emblica *	Anti-inflammatory, analgesic activity	[27]
20	*Alpinia oxyphylla *	Antiageing	[28]

**Table 2:** Evaluation of activities of protocatechuic acid in preventing different chemical carcinogenesis in rodents [30].

Species/strain/gender of animals	Carcinogen/ promoter	PCA dose/route	Target tissue	Response
F344 rats/males	4-NQO	500, 1000, and 2000 ppm/in diet	Oral cavity (tongue)/SCC	Inhibition
F344 rats/males	4-NQO	2000 ppm/in diet	Oral cavity (tongue)/SCC	Inhibition
Syrian golden hamsters/males	DMBA	200 ppm/in diet	Buccal pouch/ SCC	Inhibition
F344 rats/males	MNNG	1500 ppm/in diet	Fore stomach/SCC	No effects
F344 rats/males	AOM	1000, 2000 ppm/in diet	Colon/ADC	Inhibition
F344 rats/males	AOM	250, 500, and 1000 ppm/in diet	Colon/ADC	Inhibition
Syrian golden hamsters/males	BOP	500, 1000 ppm/in diet	Pancreas/ADC	Inhibition
F344 rats/males	DEN	500, 1000 ppm/p.o.	Liver/AD	Inhibition
A/J mice/females	NNK	1000 ppm/in diet	Lung/AD	No effects
F344 rats/males	BBN	500, 1000, and 2000 ppm/in diet	Urinary bladder/TCC	Inhibition
CD-1 mice/females	B[a]P/TPA	5, 10, 20 mM	Skin/PAP	Inhibition
ICR mice/females	DMBA/TPA	16, 160, and 1600 nM/topically to the skin 0; 40 min or 3 h before TPA	Skin/PAP	Inhibition (16 nM); enhancement of skin papilloma by 1600 nM PCA
F344 rats/males	PhIP	2000 ppm/in diet	Breast/ADC	No effects

AD: adenoma; ADC: adenocarcinoma; AOM: azoxymethane; B[a]P: benzo[a]pyrene; BBN: N-butyl-N-(4-hydroxybutyl) nitrosamine; BOP: N-nitrosobis (2-oxopropyl) amine; DEN: N-diethylnitrosamine; DMBA: 7, 12-dimethyl-benz[a]anthracene; MMMG: N-methyl-N′-nitro-N-nitrosoguanidine; NNK: 4-(methyl-nitroso-amino)-1-(3-pyridyl)-1-butanone; 4-NQO: 4-nitroquinoline oxide; PAP: squamous cell papilloma; PhIP: 2-amino-1-methyl-6-phenylimidazo[4,5-b]pyridine; SCC: squamous cell cancer; TCC: transitional cell carcinoma; TPA: 12-O-tetradecanoylphorbol 13-acetate.
